# MiR-490-5p Restrains Progression of Gastric cancer through DTL Repression

**DOI:** 10.1155/2021/2894117

**Published:** 2021-09-20

**Authors:** Jianjie Li, Xiaoyue Xu, Chunhui Liu, Xiaoxue Xi, Yang Wang, Xiaotang Wu, Hua Li

**Affiliations:** ^1^Department of Gastrointestinal Surgery, Tangshan Central Hospital, Tangshan, 06300 Hebei, China; ^2^Department of Gastrointestinal Surgery, Tangshan Gongren Hospital, Tangshan, 063000 Hebei, China; ^3^Department of General Surgery, North China University of Science and Technology Affiliated Hospital, 063000 Hebei, China; ^4^Shanghai Engineering Research Center of Pharmaceutical Translation, 200231, China

## Abstract

Gastric cancer (GC) accounts for a main cause of cancer-related deaths. This study sought for molecular mechanism of miR-490-5p/DTL axis in affecting GC progression, thus bringing new hope for treatment of GC. Expression data of differentially expressed miRNAs and mRNAs in GC tissue from TCGA database were analyzed. MiR-490-5p and DTL mRNA expression levels in GC were evaluated with qRT-PCR. Cell viability was confirmed with CCK-8 method. Cell cycle distribution and apoptosis were analyzed with flow cytometry. Cell migratory and invasive potential was proved with Transwell assay. The targeted relationship between DTL and miR-490-5p was analyzed with dual-luciferase assay. The results indicated a decreased miR-490-5p level in GC cells. MiR-490-5p upregulation hampered proliferation, migration, invasion and promote cell apoptosis. DTL was the target of and inversely associated with miR-490-5p, and it could remarkably induce the carcinogenesis of GC. MiR-490-5p mediated GC cell progression by DTL repression. In conclusion, miR-490-5p and DTL may be valuable in diagnosis and treatment for GC.

## 1. Introduction

Gastric cancer (GC) ranks fifth among all prevalent cancers worldwide and third preeminent cause of cancer-related deaths. Studies have shown that about 700,000 people die of this malignant tumor in 2018 all over the world, preceded only by lung cancer [1, 2]. Owing to insufficient specific signs for early GC, people are often diagnosed as advanced GC, with a median survival time of about 7-9 months [3]. Therefore, it is pressing to enlighten new mechanisms related to GC progression for novel and reliable approaches for diagnosis and therapy.

Numerous studies have found that overexpression or knockdown of a specific miRNA can affect the malignant progression of tumor cells via modulating expression levels of their target genes [4, 5]. MiR-490, as an important conserved RNA molecule, is theorized to be a modulator of varying human diseases (tumors) [3]. Hamfjord j and his colleagues found that miR-490-3p knockdown results in occurrence of colorectal cancer [6]. Chen and other experts manifested the repressive role of miR-490-5p in progression of renal clear cell carcinoma [7]. Besides, as Xu and other researchers proposed, miR-490-5p targets BUB1 gene, thereby hampering cell proliferation, migration and invasion of hepatocellular carcinoma (HCC) [8]. As Fang and other researchers revealed, miR-490-5p represses E2F2 and ECT2, thus hindering cell metastasis in HCC [9]. Yu and other experts suggested that miR-490-5p is an inhibitor of cell stemness in HCC through targeting ECT2 gene [10]. However, role of miR-490-5p in GC cells has not been studied.

It is illustrated by studies that miRNAs are generally controllers in cancer by targeting downstream genes. Denticleless E3 Ubiquitin Protein Ligase Homolog (DTL) gene, namely CDT2, RAMP or DCAF2, is one of DDB1 and CUL4 associated factors (DCAFs) with 7 WD40 domains at the N terminal. Besides, DTL pertains to DCAF protein family containing WD40-repeat sequence and can be used as the substrate receptor of CRL4 ubiquitin ligase [11]. At present, the modulatory role of DTL in cell cycle and DNA replication has been well elucidated [12]. For example, a study on yeast showed that lack of DTL seriously delays the progression of S phase [13]. The important role of DTL in genomic stability suggests that this ubiquitin system component may be involved in tumorigenesis [14]. It is noteworthy that numerous investigations have revealed that the level of DTL is significantly increased in many cancers [14–17]. Besides, previous studies on breast cancer and Ewing's sarcoma displayed that knockdown of DTL weakens migration and proliferation of cancer cells [16, 18]. Hiroki Kobayashi and other experts showed that overexpression of DTL leads to unfavorable outcomes in patients with GC [15]. However, study about regulatory mechanism of DTL in GC remains an unmet need.

MiR-490-5p level in GC cells was evaluated. Besides, we also explored the possible targets of miR-490-5p in GC cells and examined its capacity in GC cell biological functions. This study may generate a novel approach for clinical therapy of GC.

## 2. Materials and Methods

### 2.1. Bioinformatics Methods

Data of mature miRNAs (tumor: 446, normal: 45) and message RNAs (mRNAs) (tumor: 375, normal: 32) were downloaded from TCGA-STAD dataset of TCGA database. MiR-490-5p level was evaluated according to downloaded mature miRNA data of the TCGA-STAD dataset. The normal sample group was viewed as control, and the expression data of mRNAs were analyzed (|logFC| >2, *p*adj<0.01) with “edgeR” package for acquirement of the differentially expressed mRNAs (DEmRNAs). The target mRNAs of miR-490-5p were analyzed with bioinformatics databases TargetScan, miRDB, and mirDIP. Then, the predicted mRNAs were taken intersection with up-regulated DEmRNAs, and the mRNA with the most significant correlation with miR-490-5p was selected as the research object.

### 2.2. Cell Culture and Transfection

GC cell lines MKN-45 (BNCC337682), MGC-803 (BNCC340395) and HGC-27 (BNCC338546) and human normal gastric cell line GES-1 (BNCC337970) were provided with BeNa Culture Collection (BNCC, Shanghai, China). RPMI-1640 (BNCC341471; BNCC, China) plus 10% fetal bovine serum (FBS), penicillin (100 U/ml) and streptomycin (100 mg/ml) was used to incubate cells. The culture conditions were humid incubators with 5% CO_2_ at 37°C.

GC cells in exponential phase were harvested for transfection. Overexpressed vector of DTL (DTL), miR-490-5p mimic and their negative controls (vector and miR-NC) were provided with Shanghai GenePharma Inc. (Shanghai, China). Lipofectamine 3000 was implemented for transient transfection of plasmids or gene fragments. The cells were divided into 5 groups: (1) miR-NC; (2) miR-490-5p mimic; (3) miR-NC + vector; (4) miR-490-5p mimic+vector; (5) miR-490-5p mimic+DTL.

### 2.3. qRT-PCR

Total RNA isolation was performed with TRIzol reagent (Invitrogen, Carlsbad, USA). Regarding mRNA quantification, RevertAid First Strand cDNA Synthesis Kit (Thermo Fisher, US) was recommended for reverse transcription of RNA into cDNA. EvaGreen 2X qRT-PCR MasterMix-Low ROX (abm, Canada) was utilized for quantification of mRNA with specific primers, with GAPDH as an endogenous reference. miRNA quantification was completed with miRNA first-strand cDNA synthesis (stem-loop method) kit (Sangon Biotech, Shanghai, China) for reverse transcription. microRNA qRT-PCR kit (SYBR Green Method) (Sangon Biotech, Shanghai, China) was taken for miRNA quantification with specific primers. U6 served as an internal reference. All qRT-PCR assays were completed on Agilent Stratagene Mx3000P system (Agilent, USA). 2^-*ΔΔ*Ct^ method was used to process data. Each assay was repeated three times. Primer sequences were listed in Table 1.

### 2.4. Western Blot

RIPA lysis buffer and 1% phenyl methyl sulfonyl fluoride were utilized for extraction of total cell proteins, and the proteins were quantified by Bradford method. Next, proteins were electrophoresed (20 *μ*g per lane) by 10% sodium dodecyl sulfate polyacrylamide gel electrophoresis (SDS-PAGE). Then, they were transferred to the polyvinylidene fluoride (PVDF, Millipore) membrane. In the process of western blotting, 5% bovine serum albumin (BSA) was used to block the membrane in Tris buffered saline Tween-20 (TBS-T). Next, primary antibodies were added for cultivation at 4°C overnight. The second day, membrane was cleaned with TBS-T three times, 10 min a time, followed by addition of the secondary antibody coupled with horseradish peroxidase for 1 h of incubation at room temperature. Enhanced chemiluminescence was used to detect protein bands. Anti-GAPDH was utilized as an endogenous reference. Primary antibodies were rabbit anti-DTL and rabbit anti-GAPDH, and the secondary antibody was goat anti-rabbit IgG. All antibodies were accessed from Abcam, China.

### 2.5. Cell Proliferation Assay

CCK-8 analysis was performed. Human GC cells (1 × 10^4^) were inoculated into 96-well plates for 24, 48 and 72 h. Cell proliferation was assayed with CCK-8 kit (Dojindo, Japan). Elx800 Reader was used to record the optical density value at 450 nm.

### 2.6. Transwell Assay

GC cell migration and invasion were assessed with a modified 24-well Transwell chamber (8 *μ*m), which was pre-coated or uncoated with Matrigel. In serum-free medium, the transfected GC cells grew to 1 × 10^6^ cells/ml. Next, 200 *μ*l cell solution was supplemented to the upper chamber and medium plus 20% FBS filled into the lower chamber. 24 h later, migrating and invading cells were fixed and subjected to crystal violet for staining. Cell counting was completed in 5 random microscope fields under a microscope (100×).

### 2.7. Cell Cycle and Cell Apoptosis Analysis

After 48 h, trypsin was recommended for digestion of the harvested cells (1 × 10^6^). PBS was employed for cell rinsing with 70% ethanol for cell fixing at 4°C overnight. The second day, 500 *μ*l propidium iodide (PI)/RNase staining solution were recommended for cell resuspension and culture at 37°C for 30 min. Next, FACScan flow cytometry was implemented for detection of cell cycle distribution. To evaluate cell apoptosis, cells replicated in the cell cycle analysis were evaluated per specification of the Annexin V-FITC/PI Apoptosis Detection Kit. In short, the cells were rinsed in iced PBS and cultured in darkness with Annexin V-FITC and PI solutions for 15 min. Next, FACScan flow cytometry was employed to study cell apoptosis. No less than 10^5^ cells per sample were studied and within 1 h, they were assayed by flow cytometry.

### 2.8. Dual-Luciferase Reporter Gene Assay

First of all, 1 × 10^5^ GC cells were inoculated to 24-well plates, followed by co-transfection with 0.5 *μ*g pmirGLO vector (DTL-WT or DTL-MUT) and 20 pmol mimic and 2 *μ*l Lipofectamine 2000 reagent (Invitrogen, US). Cells were lysed 36 h later. Following protocol of Dual-Luciferase Detection Kit (Promega), luciferase activity was assayed, with luciferase activity of renilla as an internal reference.

### 2.9. Data Analysis

Data were in form of mean ± SD. Statistical analysis was completed on GraphPad Prism Version 7.0. T-test was for analysis of difference between two groups. Difference among multiple groups was analyzed by one-way ANOVA. Pearson correlation analysis was carried out to determine relationship of miR-490-5p and DTL. *P* <0.05 was considered that the difference was statistically significant.

## 3. Results

### 3.1. MiR-490-5p Level Decreased in GC Cells

**Figure 1:**
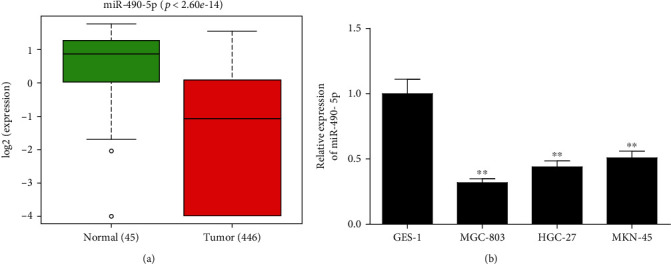
MiR-490-5p decreases in GC cell lines. A: The box plot of miR-490-5p level in normal (green) and tumor (red) groups in the TCGA-STAD; B: MiR-490-5p level in GES-1, MGC-803, HGC-27 and MKN-45 cells was assayed via qRT-PCR assay; ∗*p* <0.05, ∗∗*p* <0.01.

Numerous investigations displayed that miR-490-5p is essential in pathogenesis of a variety of cancers [7, 19, 20]. This research disclosed a remarkably decreased miR-490-5p level in GC tissue by analyzing miRNA expression in TCGA-STAD dataset (Figure 1(a)). Then, qRT-PCR was managed to analyze miR-490-5p level in normal gastric cell line and GC cell lines. Level of miR-490-5p in GC cell lines was also significantly low (Figure 1(b)). Therefore, miR-490-5p may function on carcinogenesis of GC. Next, since miR-490-5p was lowly expressed in cancer cells and its expression was relatively low in MGC-803 and HGC-27 cells, these two cell lines were selected for experiments.

### 3.2. Overexpressed miR-490-5p Hinders Progression of GC Cells

**Figure 2:**
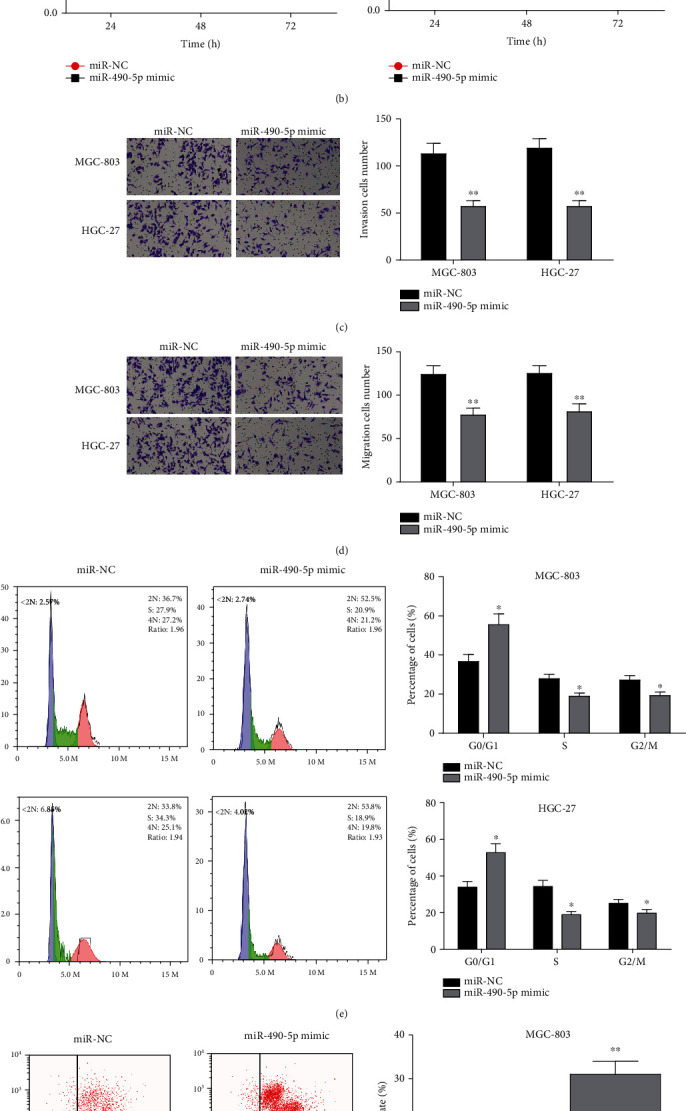
Overexpressed miR-490-5p inhibits malignant progression of GC cells. A: After transfection with miR-490-5p mimic, miR-490-5p level in MGC-803 and HGC-27 cells was significantly increased; B-D: The (B) viability, (C) migratory and (D) invasive potentials of transfected cells were detected via CCK-8 and Transwell (100×); E-F: The (E) cell cycle and (F) cell apoptosis of transfected MGC-803 and HGC-27 cells were detected via flow cytometry; ∗*p* <0.05, ∗∗*p* <0.01.

To evaluate the impact of miR-490-5p on progression of GC cells *in vitro*, MGC-803 and HGC-27 cell lines in which miR-490-5p was overexpressed were constructed. As illustrated in Figure 2(a), qRT-PCR disclosed favorable transfection efficacy in various groups. Next, CCK-8 assay measured proliferative potential of cells. It was revealed that cell proliferative potential was inhibited with overexpressing miR-490-5p (Figure 2(b)). Transwell assay exhibited that migratory potential (Figure 2(C)) and invasive potential (Figure 2(D)) of GC cells were notably down-regulated after miR-490-5p was overexpressed in comparison with the control group. Besides, flow cytometry assayed cell cycle and cell apoptosis in GC. The result showed that cell cycle was significantly stagnated at G0/G1 phase (Figure 2(e)) and apoptosis of MGC-803 and HGC-27 cell lines was promoted (Figure 2(f)) with miR-490-5p overexpression. In summary, miR-490-5p could inhibit GC cell growth and induce apoptosis *in vitro*.

### 3.3. DTL Expression Is High in GC Cells and Has an Inverse Correlation with miR-490-5p

**Figure 3:**
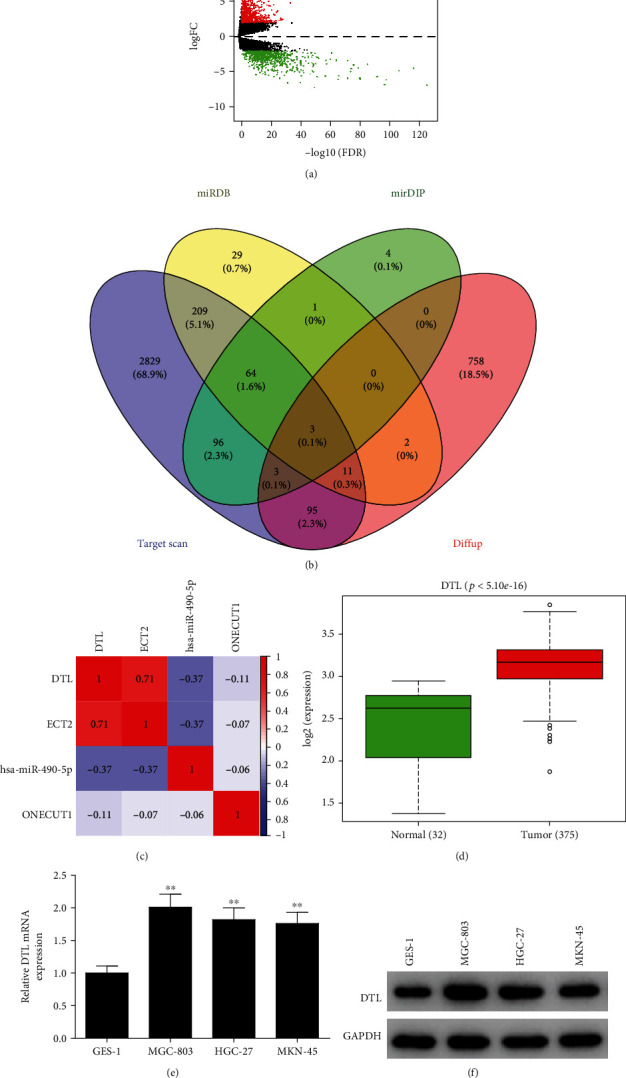
DTL is significantly highly expressed in GC cells and inversely associated with miR-490-5p. A: Volcano map of DEmRNAs in the TCGA-STAD dataset; B: Venn diagram of predicted target mRNAs of miR-490-5p and the up-regulated DEmRNAs in TCGA; C: The correlation between miR-490-5p and DTL/ECT2/ONECUT1 was analyzed with Pearson correlation analysis; D: The expression of DTL in GC tissue in TCGA; E-F: The DTL (E) mRNA and (F) protein levels in GC cells; ∗∗*p* <0.01.

Recently, much evidence has shown that miRNAs contain complementary sequences of their target mRNAs, and they can bind to the sequences to inhibit the expression of mRNAs [21, 22]. In order to check whether miR-490-5p has similar regulatory mechanism in GC, we used bioinformatics methods to predict downstream target genes of miR-490-5p. First of all, “edgeR” package was employed for differential analysis of mRNAs in GC. Thereafter, 1,641 DEmRNAs were obtained, including 872 up-regulated DEmRNAs and 769 down-regulated DEmRNAs (Figure 3(a)). Next, target gene prediction of miR-490-5p was carried out, and 3 genes were obtained, namely DTL, ECT2, and ONECUT1 (Figure 3(b)). Moreover, miR-490-5p was conspicuously negatively related to DTL and ECT2 (Figure 3(C)). It has been reported in many pieces of literature that DTL is overexpressed in multiple cancers and can promote the growth of tumor cells [23, 24]. Therefore, DTL was selected as a possible target for miR-490-5p. Besides, DTL level in the TCGA-STAD dataset was analyzed, and an increased DTL level was revealed in GC tissue (Figure 3(D)). DTL expression was assessed in GES-1 cells and GC cells. As presented in Figure 3(e)-3(f), mRNA and protein levels of DTL were noticeably upregulated in GC cells. Hence, DTL may be targeted by miR-490-5p in GC cell lines.

### 3.4. MiR-490-5p Represses DTL Level in GC Cells

**Figure 4:**
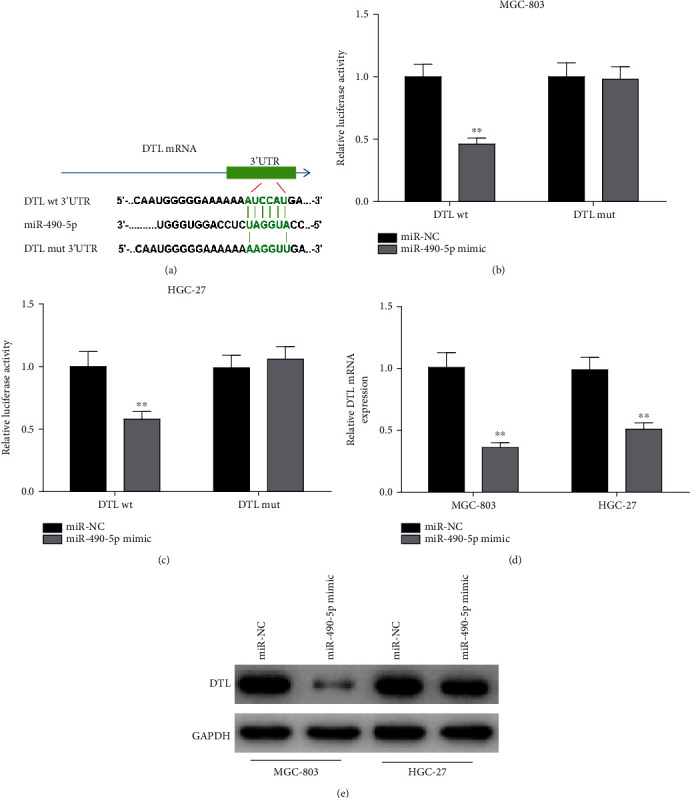
MiR-490-5p hampers DTL level in GC cells. A: Putative binding sites of miR-490-5p on DTL were predicted with bioinformatics methods; B-C: Luciferase analysis verified targeted sites; D-E: MiR-490-5p upregualtion markedly hampered (D) mRNA and (E) protein levels of DTL in MGC-803 and HGC-27 cells; ∗∗*p* <0.01.

Bioinformatics analysis predicted the binding sites between miR-490-5p and DTL, so as to verify our conjecture (Figure 4(a)). The targeted relationship was also validated via dual-luciferase assay. As suspected, miR-490-5p overexpression resulted in conspicuous decrease in luciferase activity of cells expressing DTL-WT, whereas no alternation was shown in that of cells expressing DTL-MUT (Figure 4(b), 4(C)). Besides, DTL level in GC cells was detected. It was revealed that DTL mRNA and protein levels in MGC-803 and HGC-27 cell lines were greatly decreased with overexpressing miR-490-5p (Figure 4(D), 4(e)). These findings denoted that miR-490-5p hampered DTL level in GC cell lines.

### 3.5. MiR-490-5p Hampers Proliferation, Migration, Invasion, and Promotes Apoptosis of GC Cells through Targeting DTL

**Figure 5:**
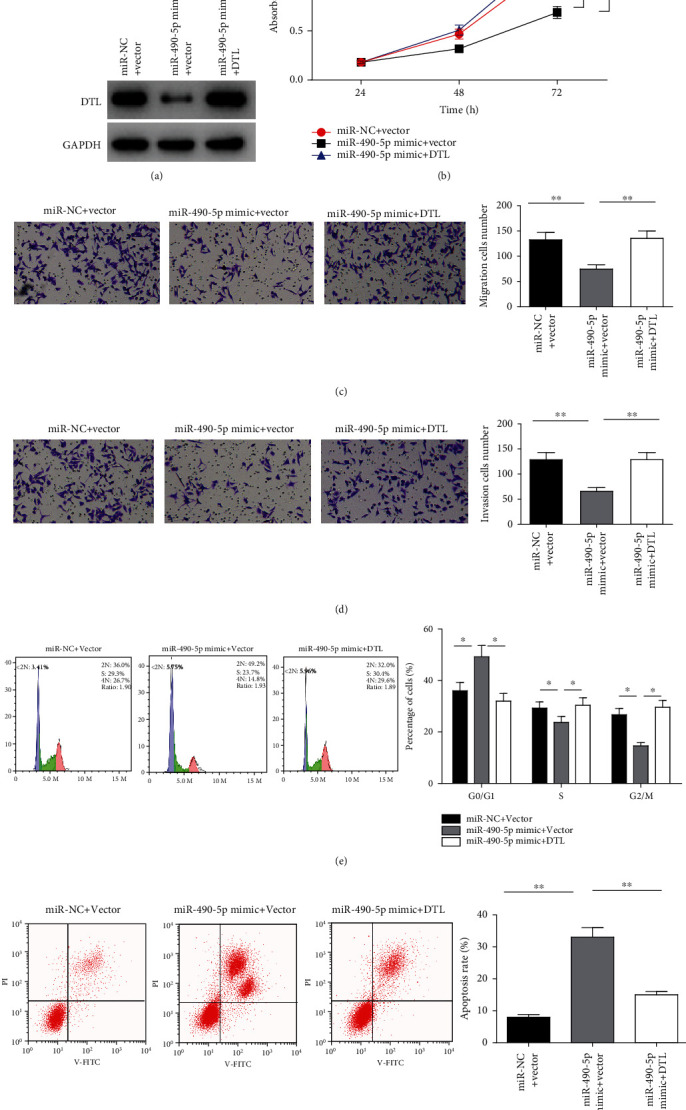
MiR-490-5p constrains cell proliferation, migration, invasion, while facilitates cell apoptosis of GC through DTL repression. A: MiR-490-5p mimic led to decrease of DTL protein level in MGC-803 cells; B: The cell viability was assessed with CCK-8 assay in miR-NC + vector, miR-490-5p mimic+vector, and miR-490-5p mimic+DTL groups; C-D: Cell (C) migration and (D) invasion were measured with Transwell assay (100 ×); E-F: The (E) cell cycle and (F) apoptosis were assayed with flow cytometry; ∗*p* <0.05, ∗∗*p* <0.01.

Next, to verify whether miR-490-5p restrains growth of GC cells via repressing DTL, functional rescue assay was conducted. Firstly, the protein level of DTL in miR-NC + vector, miR-490-5p mimic+vector and miR-490-5p mimic+DTL groups was detected by western blot, which was notably downregulated in GC cell line MGC-803 after miR-490-5p was overexpressed. However, this effect of miR-490-5p overexpression alone on DTL protein level was weakened while DTL was overexpressed concurrently (Figure 5(a)). After that, CCK-8 assay was performed to detect cell proliferative potential. The results manifested that the proliferative potential of cells was considerably hindered by overexpressing miR-490-5pin comparison to the miR-NC + vector group, while the inhibitory impact of miR-490-5p upregulation on cell proliferative potential was considerably reduced with simultaneously overexpressed miR-490-5p and DTL (Figure 5(b)). Cell migratory and invasive potentials were detected through Transwell assay and it was found that cell migratory and invasive potentials were substantially suppressed after miR-490-5p was overexpressed. However, this effect was abolished with concurrently overexpressed miR-490-5p and DTL, which was tremendously different in comparison to the miR-490-5p mimic+vector group (Figure 5(c), 5(d)). Detection of cell cycle changes and apoptotic potential in different transfection groups found that overexpression of miR-490-5p conspicuously stimulated cell cycle arrest in G0/G1 Phase as well as cell apoptosis, while simultaneous overexpression of miR-490-5p and DTL markedly attenuated the arresting effect on cell cycle and the promotion on cell apoptosis (Figure 5(e), 5(f)). It was indicated that miR-490-5p might constrain cell progression of GC and promote cell apoptosis via DTL repression.

## 4. Discussion

In recent years, GC diagnosis and treatment have been greatly improved and developed [25, 26]. However, the poor prognosis of GC makes it one of the most prevalent fatal diseases. Therefore, new treatment approaches are in high demand to provide more treatment options for GC patients [27].

Accumulating investigations manifested that miRNA takes a pivotal part in a variety of cancers. MiR-490-5p was found to be significantly down-regulated in GC cells. Proliferation, migration, and invasion of GC cells were remarkably hindered while cell apoptosis was promoted after miR-490-5p was overexpressed. MiR-490 plays different roles in different cancers. Chen and other experts declared that miR-490-5p is lowly expressed in the tissue and cells of clear cell renal cell carcinoma (ccRCC), and it constrains the growth of ccRCC via PIK3CA [7]. MiR-490-3p decreases in colorectal cancer and GC [3, 28]. It is reported that miR-490 also decreases in esophageal cancer, pancreatic cancer and other digestive cancers [29]. According to Li and other colleagues, miR-490-5p upregulation leads to inhibition on proliferation and induction on cell cycle arrest in G0/G1 phase in bladder cancer [30]. We illustrated that miR-490-5p exerted a similar effect in GC, which hindered malignant progression of the disease. These findings are in accordance with previous studies.

As one of the DCAFs of CUL4A (Cullin 4A), DTL takes a vital part in cell cycle and DNA repair. DTL on chromosome 1q32.1-32.2 encodes a putative 730-amino acid protein containing 6 highly conserved WD40-repeat domains [18]. DTL is key in cell cycle arrest, progression of GC, hepatocellular carcinoma, rhabdomyosarcoma, and breast cancer [24, 31–34]. As Cui and other researchers found, DTL facilitates cell mobility and proliferation of cancer through degradation of PDCD4, thus promoting the progression of cancer cells [23]. Alexander Baraniskin and other experts found that DTL is upregulated in colorectal cancer cells, besides, miR-30a-5p constrains colorectal cancer growth through targeting DTL [24]. However, the regulatory mechanism of DTL in GC has not been studied. In this study, there was a negative correlation between the expression of DTL and that of miR-490-5p, which was validated through bioinformatics prediction and experiments. Rescue assays ascertained that the modulatory role of miR-490-5p on malignant progression of GC was achieved through DTL repression partially. These results are consistent with those illustrated by other researchers on function of DTL and have great significance for the targeted therapy of GC.

In conclusion, our current results reveal that miR-490-5p functions as a tumor repressive gene that hampers malignant progression of GC and can regulate the expression of DTL. More importantly, the miR-490-5p/DTL axis will contribute to developing new therapeutic targets for GC. In addition, our data imply that miR-490-5p is potential to become a biomarker for diagnosis and prognosis of GC. Nonetheless, the main weakness of this study was the large difference between the number of normal samples and tumor samples. Hence, we will collect clinical tissue for verification. We will investigate the upstream modulatory mechanism of miR-490-5p and the downstream signaling pathway of DTL for better validating miR-490-5p as a biological target for GC.

## Figures and Tables

**Table 1:** 

Gene	Sequence	
miR-490-5p	Forward	5'-CATGGATCTCCAGGTGG-3'
	Reverse	5'-TGGTGTCGTGGAGTCG-3'
U6	Forward	5'-CTCGCTTCGGCAGCACA-3'
	Reverse	5'-AACGCTTCACGAATTTGCGT-3'
DTL	Forward	5'-AGGCAAAGAGAATAGTTCCCCAG -3'
	Reverse	5'-GGACTTCGTGGAGATGGA-3'
GAPDH	Forward	5'-GGACCTGACCTGCCGTCTAG-3'
	Reverse	5'-GTAGCCCAGGATGCCCTTGA-3'

## Data Availability

The data and materials in this current study are available from the corresponding author on reasonable request.
